# Lipoprotein(a)—clinical aspects and future challenges

**DOI:** 10.1007/s11789-015-0075-z

**Published:** 2015-03-03

**Authors:** Bilgen Kurt, Muhidien Soufi, Alexander Sattler, Juergen R. Schaefer

**Affiliations:** Internal Medicine, Preventive Cardiology, University Clinic Gießen and Marburg, 35033 Marburg, Germany

**Keywords:** Lipoprotein(a), Lp(a), Coronary vascular disease, Prevention, Lipid lowering therapy, Lipoprotein(a), Lp(a), koronare Herzerkrankung, Prävention, lipidsenkende Therapie

## Abstract

Lipoprotein(a) (Lp(a)) was first described by K. Berg and is known for more than 50 years. It is an interesting particle and combines the atherogenic properties of low-density lipoprotein (LDL)-cholesterol as well as the thrombogenic properties of plasminogen inactivation. However, due to technical problems and publication of negative trials the potential role of Lp(a) in atherosclerosis was severely underestimated. In recent years our understanding of the function and importance of Lp(a) improved. Interventional trials with niacin failed to demonstrate any benefit of lowering Lp(a); however, several studies confirmed the residual cardiovascular disease (CVD) risk of elevated Lp(a). LDL/Lp(a) apheresis is able to lower Lp(a) and some new drugs under development should help us to lower Lp(a) in the near future. It will be important to follow this with hard endpoint trials. Until then most clinicians recommend the use of an aggressive LDL-lowering approach in patients with high Lp(a). Since most of these patients with high Lp(a) might have manifested atherosclerosis anyway, we would also consider the use of acetylsalicylic acid.

## Abstract (de)

Lp(a) wurde erstmals von K. Berg beschrieben und ist seit mehr als 50 Jahren bekannt. Es ist ein interessanter Lipoproteinpartikel und kombiniert die atherogenen Eigenschaften des LDL-Cholesterins mit den thrombogenen Eigenschaften einer gestörten Plasminogen Aktivierung. Leider wurde die potentielle Bedeutung des Lp(a) Spiegels als kardiovaskulärer Risikofaktor aufgrund technischer Probleme bei dessen Messung und der Publikation einer Reihe negativer Studien über lange Zeit hinweg unterschätzt. In den letzten Jahren hat sich unser Verständnis bezüglich der Funktion und Bedeutung von Lp(a) für die Atherosklerose deutlich gebessert. Medikamentenstudien mit der Nikotinsäure zeigten trotz einer Senkung des Lp(a) keinen Nutzen, dagegen wurde die Bedeutung von erhöhtem Lp(a) für die Atherosklerose in mehreren Studien bestätigt. Die LDL/Lp(a) Apherese ist in der Lage Lp(a) deutlich zu senken. Zudem sind einige neuere Medikamente in Entwicklung, die ebenfalls Lp(a) senken können. Hier wird es wichtig sein, deren Effekte auch im Rahmen von Studien mit harten klinischen Endpunkten zu überprüfen. Bis dahin werden die meisten Ärzte bei Ihren Patienten mit hohem Lp(a) eine intensivierte LDL senkende Therapie durchführen sowie additive Risikofaktoren entsprechend angehen. Da die meisten der Patienten mit hohem Lp(a) Spiegel ohnehin bereits eine manifeste Atherosklerose haben dürften, sollte auch über den Einsatz von Acetylsalicylsäure nachgedacht werden.

## Introduction

The story of lipoprotein(a) (= Lp(a)) is remarkable and troubled over the past decades since its discovery by Berg in the year 1963 [1]. In the meantime high serum Lp(a) level is a well-recognized and established risk factor for cardiovascular disease (= CVD) with a causal relation of Lp(a) genotype and Lp(a) level. However due to technical problems in measuring Lp(a) there were some hurdles in recent years in understanding the real importance of Lp(a) as a cardiovascular disease (CVD) risk factor. Unfortunately a series of powerful and well-published studies on Lp(a) and its role for CVD and stroke were negative—most likely for technical reasons such as freezing the samples prior to Lp(a) measurement, cross reactivity of the assay, acute phase reaction, and others [2–4]. These studies lowered research interest in past years, despite the fact that other excellent lipidologists identified Lp(a) as an independent CVD risk factor [5]. The former technical problems of measuring Lp(a) precisely in the old but well-published studies still have an impact on several follow-up publications performing meta-analysis of long-term prospective studies that recorded Lp(a) and vascular morbidity and/or mortality by which they found only a modest association of Lp(a) concentration with risk of coronary heart disease (CHD) and stroke [6]. Interestingly enough, due to the ease of analysis, there were mostly genetic studies which reintroduced sight of and interest in Lp(a) as an independent CVD risk factor [7]. Clarke et al. demonstrated by the PROCARDIS cohort that two LPA variants (rs10455872 and rs3798220) were associated with an increased level of Lp(a) lipoprotein but also an increased risk of coronary disease. By this they concluded that their findings indicate a causal role of Lp(a) lipoprotein in coronary artery disease (CAD) [8]. Schunkert et al. performed a meta-analysis of 14 genome-wide association studies of CAD comprising 22,233 individuals with CAD and 64,762 controls followed by genotyping of top association signals in 56,682 additional individuals. By this Schunkert et al. were able to identify 13 loci newly associated with CAD and confirmed the association of 10 of 12 previously reported CAD loci, including rs3798220 of Lp(a) [9].

## Structure and function of Lp(a)

**Figure 1:**
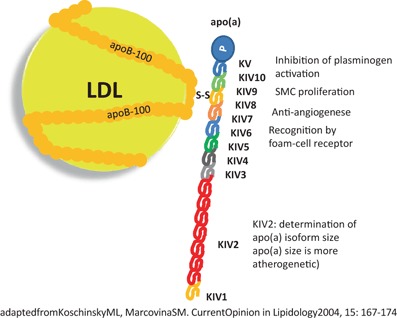

Lp(a) is basically a composite structure of low-density lipoprotein (LDL) which binds by a disulfide bridge to an additional glycoprotein, the so-called apolipoprotein(a) (= apo(a)). Both together, the LDL and apo(a) form the Lp(a) particle (Fig. 1) [10]. The apo(a) protein part has a total of five cysteine-rich domains which are called “kringles” with the fourth kringle being homologous with plasminogen [11]. Plasminogen is a protein which—once activated by tissue plasminogen activator (tPA) to plasmin—is able to resolve blood clots. Apo(a) is able to interfere with plasminogen activation, which inhibits thrombolysis [12]. Taken together, Lp(a) is a highly complex structure. By the LDL part it is potentially atherogenic; by the apo(a) part it is potentially thrombogenic.

## Lp(a) measurement

For Lp(a) “particle number” as a molar concentration is a better CVD risk predictor compared to “component-based metrics.” Therefore there is need for a mass-insensitive Lp(a) assay, and in future studies, the Lp(a) measurements should be performed with comparable and stable test systems [13]. More importantly, currently it is unclear when to measure Lp(a) at all. Some physicians deny the measurement not only for costs but also for the lack of treatment options to lower Lp(a). Kostner et al. see that the consensus reports of scientific societies are currently still prudent in recommending the measurement of Lp(a) routinely for assessing CVD risk. For example, the present European consensus statement recommends Lp(a) measurement in subjects with a 10-year risk of fatal CVD 3 % and more [14]. They see this mainly due to the lack of definite intervention studies demonstrating that lowering Lp(a) reduces hard CVD endpoints, a lack of effective medications for lowering Lp(a), the highly variable Lp(a) concentrations among different ethnic groups, and the challenges associated with Lp(a) measurement [15]. In their excellent review, Kostners and März recommend the measurement of Lp(a) in all these patients with premature CVD and premature stroke. However, they also recommend Lp(a) measurement in patients who fall into an intermediate risk group when classical risk algorithms are used such as the Framingham risk score, the PROCAM risk score, the ESC Heart Score, or the Australian and New Zealand risk calculator. The rationale for this is the fact that patients with high Lp(a) might get restratified into a higher risk category. Since there is no treatment option to lower Lp(a) in these patients, treatable risk factors such as LDL cholesterol, hypertension, smoking, diabetes, and obesity, should be treated intensively. Kostner et al. also recommend Lp(a) measurement in patients with (1) recurrent or rapidly progressive vascular disease despite being on lipid-lowering medication; (2) familial hypercholesterolemia (FH) or other forms of genetic dyslipidemias; (3) low high-density lipoprotein cholesterol (HDL-C); (4) genetic defects related to hemostasis and homocysteine; (5) diabetes mellitus; (6) renal disease; and (7) autoimmune diseases.

## Lp(a) in vivo kinetics

The first in vivo turnover studies of Lp(a) were done by Krempler and Kostner more than 30 years ago [16]. They injected radiolabeled Lp(a) (^125^Iodine) into nine volunteers with plasma concentrations ranging from 5 to 75 mg/dL. By following the decay curve of the specific radioactivity over time they found that Lp(a) concentrations correlated with the production rate, while there was no correlation with Lp(a) residence times. However in certain disease states, such as chronic renal failure, Lp(a) in vivo metabolism differs from normal. By utilizing a stable isotope tracer technique (3Deuterium-labeled L-Leucine) we found normal production rates of Lp(a) in patients with renal failure undergoing hemodialysis. However the Lp(a) residence time was twice as long as normal. This finding explains the elevated Lp(a) levels in hemodialysis patients as a result of an impaired catabolism [17]. The reports of the side of Lp(a) production and assembly remain controversial [18]. There are technical challenges to isolate the pure apoB-100 protein part solely from the Lp(a) particle and to avoid—among others—any contamination with apoB-100 from the LDL particles. To answer this question, endogenous labeling of both proteins, the apo(a) as well as the apoB part of the Lp(a) particle, in addition to apoB from very low-density lipoprotein (VLDL), intermediate-density lipoprotein (IDL), and LDL is an appropriate approach. For this we performed in vivo turnover studies utilizing 3Deuterium-labeled L-Leucine as a stable isotope tracer in a total of nine subjects. This approach labels all proteins and the investigator needs simply to isolate the individual proteins of interest and measure the tracer enrichment within this protein by mass spectrometry [19]. By this we found similar mean production rates (PRs) for the proteins of Lp(a), namely apo(a) (1.15 nmol/kg/d) as well as for apoB-100 (1.31 nmol/kg/d) both isolated from Lp(a). These were significantly different from the PR for apoB-100 isolated from LDL, 32.6 nmol/kg/d. Also mean fractional synthetic rate (FSR) and residence time (RT) values for Lp(a)–apo(a) were similar to those of Lp(a)–apoB and different from those for LDL–apoB. From these studies we were able to conclude that there are two different kinetic apoB pools within Lp(a) and LDL. This again suggests intracellular (or close to the cellular membrane) Lp(a) assembly from apo(a) and newly synthesized apoB-100 of LDL. According to our data there is no way that apo(a) could assemble with secreted LDL particles later on in the blood [20]. This information is crucial for potential treatment approaches which might focus on the assembling of the Lp(a) compounds. However there are also conflicting in vivo turnover studies where the tracer enrichment of apoB from Lp(a) was comparable to that of LDL apoB. This might be due to differences of the study protocol such as fasting versus fed status; however technical problems such as minor LDL apoB contaminations might also have an influence and could easily generate conflicting results. Notably small LDL apoB contaminations, which can easily happen, might affect the apoB kinetics of Lp(a) seriously. Therefore we isolated Lp(a) as well as LDL by ultracentrifugation followed by sodium dodecyl sulfate polyacrylamide gel electrophoresis (SDS PAGE) separation of apo(a) and apoB-100, respectively.

## Lp(a) in renal disease

It is well known that patients with chronic renal disease have elevated Lp(a). However, the reason for this is not clear. Kostner et al. suggested a decrease in Lp(a) catabolism in patients with renal dysfunction since they found a decrease of urinary apo(a) excretion [21]. In order to clarify Kostner’s observation we performed in vivo kinetic studies in hemodialysis patients utilizing stable isotope labeling of lipoproteins including Lp(a) (see section “Lp(a) in vivo kinetics” above). By this we were able to confirm that elevated Lp(a) in renal disease is due to a delay of Lp(a) metabolism [22]. As a matter of fact the kidney appears to play a major role in Lp(a) catabolism.

## LDL/Lp(a) apheresis

Until now the only highly effective approach to lower Lp(a) by more than 50 % is LDL/Lp(a) apheresis. However this is an invasive, costly, and time-consuming approach, which requires mostly weekly treatments. Therefore it is important that this approach proves to be safe and effective. For this Jaeger et al. studied 120 patients on LDL apheresis and found a significant reduction of CAD events under LDL apheresis compared to the time before this treatment [23]. In addition most recently Leebmann et al. performed the Pro(a)LiFe study as a prospective observational multicenter trial to study the effect of chronic lipoprotein apheresis on cardiovascular events in 170 patients undergoing LDL apheresis to lower Lp(a). They found a clear reduction of events in a timeframe of 2 years under treatment compared to the same time before LDL apheresis [24]. Despite some problems due to the study design, these data suggest beneficial effects of LDL apheresis, especially by lowering Lp(a) in addition to LDL cholesterol (Tables 1, 2, 3).

**Table 1:** 

Gene	Effect on Lp(a) level (maximum reported value)
Apo(a)	Up to 90 %
LDL-R	Two- to threefold increase
MODY (HNF-4a)	3.3-fold increase

**Table 2:** 

Nongenetic factors	Effect on Lp(a) level (maximum reported value)
Acute phase	Up to twofold increase
Renal disease	Threefold increase
Liver diseases	Up to 90 % reduction
Alcohol	Up to 20–57 % decrease

**Table 3:** 

Hormone	Effect on Lp(a) level (maximum reported value)
Thyroxine	10–25 % reduction
Pregnancy	2.5–3-fold elevation
Estrogens	37 % reduction
Progesterone	3–5 % reduction
Tamoxifen	35 % reduction
Tibolone	35 % reduction
Raloxifene	18 % reduction
Testosterone	30–40 % reduction
Anabolic steroids	60–70 % reduction
ACTH	30–40 % reduction

## Treatment to lower Lp(a)

The treatment of elevated Lp(a) is troublesome since we have no real good drug treatment available to lower elevated Lp(a) (Table 4). The only established Lp(a)-lowering treatment is the use of nicotinic acid, fibrates, or performing LDL/Lp(a) apheresis. In addition there are new classes of upcoming—or currently studied—therapeutic agents with some Lp(a)-lowering effect, such as apoB antisense oligonucleotides, apo(a) antisense oligonucleotides, microsomal triglyceride transfer protein inhibitors, cholesterol ester transfer protein inhibitors, and proprotein convertase subtilin/kexin type 9 (PCSK9) inhibitors.

**Table 4:** 

Treatment	Effect on Lp(a) level (maximum reported value)
Nicotinic acid	30–35 % reduction
Bezafibrate	Up to a 39 % reduction
Statins	Inconsistent
L-Carnitine	10–20 % reduction
N-acetyl-cysteine	Controversial
Acetylsalicylic acid	10–20 % reduction
LDL/Lp(a) apheresis	50–80 % reduction

The use of statin treatment alone is not able to reduce the risk of elevated Lp(a), which is an important residual risk factor also under lipid lowering therapy with potent statins. Khera et al. showed with the JUPITER study in 9612 participants that baseline Lp(a) level was an important residual risk for CVD. The JUPITER trial (justification for the use of statins in prevention: an intervention trial evaluating rosuvastatin) studied the effect of rosuvastatin 20 mg/d or placebo in a primary prevention trial. In JUPITER the baseline Lp(a) concentrations were associated with incident cardiovascular disease (HR 1.18; 95 % confidence interval, 1.03–1.34; *P* = 0.02) and the on-statin-treatment Lp(a) concentrations demonstrated a residual risk of cardiovascular disease (HR 1.27; 95 % confidence interval, 1.01–1.59; *P* = 0.04). This again was independent of LDL-cholesterol or other factors [26]. This demonstrates that Lp(a) is a CVD risk factor beyond LDL cholesterol and needs our full attention above lowering LDL cholesterol alone.

PCSK9 monoclonal antibody treatment is able to lower Lp(a) significantly for up to 32 % in subjects with hypercholesterolemia receiving statin therapy [27]. The Lp(a)-lowering effect was confirmed in a pooled analysis of data from 1359 patients on evolocumab, a fully human monoclonal antibody to PCSK9 [28]. However the mechanism for this Lp(a)-lowering effect by monoclonal antibody to PCSK9 is currently unclear. Studies in homozygote FH patients with no LDL receptor (LDLR) expression showed an LDLR-unrelated decrease in Lp(a), indicating that Lp(a) catabolism is not driven primarily by the LDLR. At this point it is important to mention the fact that Lp(a) binds to two other related receptors in the LDLR family, VLDL receptor (VLDLR) and megalin/330gp, with higher affinity than LDLR or LDLR-related protein (LRP). The VLDLR is expressed in heart, skeletal muscle, macrophages, and adipose tissue, but not in the liver [29]. Interestingly enough Roubtsova et al. could show that VLDLR is regulated in part by endogenous PCSK9 as well. They found in their PCSK9(−/−) mice model a 40-fold higher cell surface level of VLDLR and an accumulation of 80 % more visceral adipose tissue compared to wild mice. Roubtsova et al. conclude that PCSK9 is pivotal in fat metabolism by maintaining high circulating cholesterol levels due to an increase of hepatic LDLR degradation and limiting visceral adipogenesis by regulating the adipose VLDLR [30]. Taking these studies together we would speculate that the decrease of Lp(a) under PCSK9 antibody treatment can be explained by an increased binding of Lp(a) to the upregulated VLDLR. Our hypothesis would also explain that Lp(a) can even be lowered by PCSK9 antibody treatment in homozygous FH patients. Stein et al. studied eight patients with LDLR-negative (*n* = 2) or LDLR-defective (*n* = 6) homozygous FH. They were treated with subcutaneous 420 mg evolocumab every 4 weeks for ≥ 12 weeks, followed by 420 mg evolocumab every 2 weeks for an additional 12 weeks. In the six LDLR-defective FH patients they found an Lp(a) decrease of 10 % and a LDL cholesterol decrease of 22,9 %. However in the two LDLR-negative FH patients they found a Lp(a) decrease of up to 16.8 % but no decrease in LDL cholesterol [31]. The study by Stein et al. is limited by the small number of patients since homozygote FH is a very rare disease. Nevertheless we see that—in contrast to lowering LDL—the Lp(a)-lowering effect of PCSK9 antibody treatment is LDLR independent. From this we speculate that Lp(a) lowering under PCSK9 inhibition results from the interaction with other receptors – such as the VLDLR. Since the VLDLR is also found on the macrophages this mechanism could also lead to an undesired accumulation of Lp(a) within macrophages. Therefore the true mechanism of the Lp(a)-lowering effect needs to be studied in more detail.

Other drugs such as lomitapide and mipomersen are also able to lower Lp(a) slightly. Lomitapide is an inhibitor of microsomal triglyceride transfer protein (MTP). MTP is necessary for assembling apoB-containing lipoproteins. Cuchel et al. studied in 29 patients with homozygous FH the effect of lomitapide and found a decrease of plasma Lp(a) levels by 15 % [32]. Mipomersen, is a small oligonucleotide which inhibits apoB production by interfering with apoB mRNA. Raal et al. studied 45 homozygous FH patients under treatment with mipomersen and found a reduction of plasma Lp(a) levels by 31 % [33]. Merki et al. studied whether an antisense oligonucleotide (ASO) directed to apo(a) would be able to reduce apo(a) and Lp(a) levels in a transgenic mouse model. As a matter of fact their ASO reduced Lp(a) by up to 86 % in different mice models [34]. These studies demonstrate that both, apo(a) synthesis as well as apoB synthesis, regulate Lp(a) particle assembly and secretion [35].

However, lowering Lp(a) levels alone is not enough. We need to reduce hard clinical endpoints by lowering Lp(a); as a matter of fact we need endpoint studies with Lp(a)-lowering drugs. So far nicotinic acid (2–4 g/day) was considered the most effective therapy to reduce Lp(a) by as much as 38 %. In addition, nicotinic acid lowers LDL cholesterol, apo B-100, and triglycerides, and raises HDL cholesterol. However, most recently the so-called AIM-HIGH (atherothrombosis intervention in metabolic syndrome with low HDL/high triglyceride and impact on global health outcomes) trial ended without any clinical benefit. AIM-HIGH had a treatment arm randomized to simvastatin plus placebo or simvastatin, plus extended-release niacin (ERN, 1500–2000 mg/day). The on-treatment LDL cholesterol was rather low and was within the range of 40 to 80 mg/dL. Albers et al. reported that Lp(a) levels were predictive of CV events in both simvastatin plus placebo and the simvastatin plus ERN group. However, despite the fact that ERN decreased Lp(a) by 21 % there was no reduction of CV events. The authors conclude that (1) Lp(a) is associated with increased CV risk in both treatment groups indicating that it contributes to residual CV risk; and (2) there is no evidence that ERN is able to reduce CV risk despite lowering Lp(a) [36]. This finding underlines that we need hard endpoint data to prove the Lp(a)-lowering concept and that the Lp(a)-lowering mechanisms (i.e., delay in production or increase of catabolism) might be crucial.

## Acetylsalicylic acid

Since the mechanism of atherosclerosis by elevated Lp(a) involves both LDL effects as well as thrombotic effects due to plasminogen interaction, it might be wise to consider antithrombotic treatment approaches, especially in patients with high Lp(a). Chasman et al. studied the genotypes of rs3798220 in 25,131 initially healthy Caucasian subjects of the Women’s Health Study [37]. Median Lp(a) levels at baseline were 10.0, 79.5, and 153.9 mg/dL for major allele homozygotes, heterozygotes, and minor allele homozygotes, respectively (*P* < 0.0001). Chasman et al. found during 9.9 years of follow-up that subjects with the minor allele (3.7 %) had a twofold higher risk of major cardiovascular events than noncarriers (HR = 2.21, 95 % confidence interval, CI: 1.39–3.52). Interestingly the treatment with acetylsalicylic acid reduced the risk more than twofold (HR = 0.44, 95 % CI: 0.20–0.94). If these data should be confirmed by other trials, acetylsalicylic acid might be an important nonlipid approach to lower CVD risk in patients with high Lp(a) levels.

## Summary

In summary we would recommend measurement of Lp(a) in patients with intermediate or high CVD risk, rapid and unclear progression, and/or premature CVD or stroke. However the practical consequences are limited since until now we have no established and endpoint-proven Lp(a)-lowering therapy. The use of niacin is questionable in light of the AIM-HIGH trial and the use of acetylsalicylic acid needs to be proven by other trials as well. Clearly the current approach of a more aggressive LDL-lowering therapy in patients with high Lp(a) seems to be rational but also needs confirmation by hard-endpoint studies.
